# Isolated and Concomitant Tricuspid Valve Replacement: Long-Term Survival and Predictors of Mortality in a 25-Year Cohort

**DOI:** 10.31083/RCM38102

**Published:** 2025-11-11

**Authors:** Yilin Pan, Linqi Liu, Xiaozheng Zhou, Jiyuan Luo, Yu Wang, Jiawei Li, Xiubin Yang, Bin Mao, Kun Hua

**Affiliations:** ^1^Department of Cardiac Surgery, Beijing Anzhen Hospital, Capital Medical University, 100029 Beijing, China; ^2^Department of Cardiology, Beijing Anzhen Hospital, Capital Medical University, 100029 Beijing, China; ^3^National Clinical Research Center for Cardiovascular Diseases, Beijing Anzhen Hospital, Capital Medical University, 100029 Beijing, China; ^4^School of Nursing, Capital Medical University, 100069 Beijing, China

**Keywords:** tricuspid regurgitation, tricuspid valve replacement, long-term outcomes, morbidity and mortality

## Abstract

**Background:**

Tricuspid valve replacement (TVR), particularly as an isolated procedure, is historically associated with high perioperative risk and poor outcomes. This study aimed to evaluate in-hospital and long-term outcomes of isolated versus concomitant TVR and identify predictors of morbidity/mortality in patients with severe tricuspid regurgitation (TR).

**Methods:**

This retrospective study included 245 consecutive adult patients who underwent surgical TVR at Beijing Anzhen Hospital between 1993 and 2019. Primary outcomes were in-hospital mortality and long-term survival. Univariate and multivariate logistic regression analyses were conducted to determine factors associated with in-hospital mortality, adjusting for chronic kidney disease (CKD) and TRI-SCORE. Additionally, univariate and multivariate Cox regression analyses were performed to identify factors associated with long-term mortality, adjusting for age, CKD, TRI-SCORE, and previous cardiac surgery history. Propensity score matching (PSM) and inverse probability of treatment weighting (IPTW) were utilized to adjust for baseline differences.

**Results:**

Patients were categorized into two groups: isolated TVR (n = 128) and concomitant TVR (n = 117). The mean age was 47 ± 13 years, 58.4% were male, and the mean left ventricular ejection fraction was 62 ± 10%. Isolated TVR patients had lower in-hospital mortality (7.8% vs. 17.9%; *p* = 0.017) compared to concomitant TVR patients. At 1, 5, and 10 years, the survival rates for isolated TVR were 89.1%, 83.3%, and 77.7%, respectively. For concomitant TVR, the corresponding rates were 72.6%, 68.9%, and 60.5%, respectively. Multivariate analysis identified isolated TVR as protective against in-hospital death (odds ratio (OR) = 0.40, 95% confidence interval (CI): 0.17–0.95; *p* = 0.037) and overall mortality (hazard ratio (HR) = 0.49, 95% CI: 0.30–0.81; *p* = 0.005). Additionally, TRI-SCORE and CKD were associated with in-hospital mortality, and both remained significant predictors of long-term mortality. IPTW and PSM analyses confirmed the results.

**Conclusions:**

Isolated TVR is associated with lower in-hospital and long-term mortality compared to concomitant TVR. Early referral before multivalve disease progression and meticulous patient selection—particularly avoiding advanced right-sided heart failure or renal dysfunction—may optimize outcomes. These findings advocate for timely isolated TVR in select TR patients to mitigate the compounding risks of delayed intervention.

## 1. Introduction

Tricuspid regurgitation (TR) is a significant valvular heart condition that often coexists with other cardiac diseases, yet it has historically received less clinical attention compared to left-sided valve disorders [1]. Despite its prevalence in the general population, TR often remains underdiagnosed and undertreated [2]. Evidence indicates that severe TR is an independent risk factor for long-term mortality [3, 4, 5, 6]. Management of TR remains a subject of ongoing debate, particularly regarding the optimal timing and appropriateness of surgical intervention. Some experts question the benefits of surgery on long-term survival [7], while others argue that earlier intervention can yield better patient outcomes [8]. This debate is driven in part by the heterogeneity of TR itself (functional versus organic), the wide spectrum of patient comorbidities, and the variability in study populations, all of which contribute to inconsistent findings in the literature.

Surgical procedures targeting the tricuspid valve (TV) are relatively uncommon and are most often performed in conjunction with interventions on other valves [1]. Tricuspid valve replacement (TVR) is even more rarely performed, accounting for less than 5% of all valvular surgeries, and is associated with significant morbidity and mortality, with a constant in-hospital mortality rate of around 10% [9, 10]. Historically, isolated TVR has been considered high-risk due to the advanced disease state and comorbid conditions present at the time of surgery [11, 12]. Differences in patient demographics, underlying comorbid conditions, causes of TR, and the combined reporting of TV repair and replacement lead to inconsistent information regarding TVR. As a result, reliable data on the precise risks, postoperative outcomes, and ideal timing for TV surgery remain scarce.

This study aims to evaluate and compare in-hospital and long-term outcomes between isolated and concomitant tricuspid valve replacement, and to identify factors influencing morbidity and mortality in these patients. Additionally, the analysis highlights the importance of early surgical intervention and careful patient selection, emphasizing the need to optimize clinical decision-making to improve prognosis in tricuspid valve surgery.

## 2. Methods

### 2.1 Study Design and Patient Population

This retrospective study analyzed consecutive patients who had severe TR and underwent TVR surgery at Beijing Anzhen Hospital between November 1993 and February 2019. Decisions regarding the need for valve replacement and choice of prosthesis were made intraoperatively by the attending surgeon, with a strong institutional preference for valve repair whenever feasible. The inclusion criteria comprised patients with severe TR who underwent isolated or concomitant TVR and had complete preoperative and postoperative clinical data. Exclusion criteria included patients under 18 years of age, those with missing or incomplete clinical data, and individuals undergoing surgery for congenital tricuspid valve disease, as the conditions represent distinct clinical scenarios with unique preoperative profiles and outcomes that are typically treated as separate categories in the literature [1]. Patients who underwent TVR without additional major interventions (e.g., other valve surgery or coronary artery bypass grafting) comprised the isolated-TVR cohort. The primary endpoint was long-term all-cause mortality, while secondary endpoints included in-hospital morbidity and complications such as stroke, renal failure, hepatic failure, bleeding and reoperation. This study was approved by the Ethics Committee of Beijing Anzhen Hospital, Capital Medical University (approval number: 2020101X). Due to the retrospective nature of the study and the use of deidentified patient data, the requirement for informed consent was waived. Data supporting the findings of this study are available from the corresponding author upon reasonable request.

### 2.2 Data Collection and Outcome Measurements

Data were extracted from electronic medical records and included demographic information, preoperative characteristics, intraoperative details, and postoperative outcomes. Right-sided heart failure signs were defined as the presence of clinical symptoms such as peripheral edema, hepatomegaly, spleen enlargements and ascites. The TRI-SCORE was calculated for each patient using the original criteria described by Dreyfus *et al*. [13] In-hospital mortality was defined as all-cause death occurring during the initial hospitalization. Stroke was defined as a persistent central neurologic deficit, assessed by a neurologist. Acute renal failure was defined as an increase in serum creatinine by ≥0.3 mg/dL within 48 hours or a 1.5-fold increase from baseline, or urine output <0.5 mL/kg/h for 6 hours [14]. Liver failure was defined as the presence of coagulopathy (INR >1.5) and any degree of encephalopathy in a patient without pre-existing cirrhosis or as acute decompensation in a patient with chronic liver disease [15]. Re-exploration for bleeding was defined as any return to surgery for bleeding within the first 24 postoperative hours.

All patients received postoperative anticoagulation according to valve type and evolving institutional guidelines. Mechanical-valve recipients were started on warfarin targeting an INR of 2.5–3.5, with monthly monitoring at dedicated clinics; aspirin (75–100 mg/day) was added for those with atrial fibrillation or prior thromboembolism. Bioprosthetic-valve recipients received warfarin for 3–6 months (INR 2.0–3.0) before switching to lifelong aspirin, unless contraindicated. Follow-up was completed by scheduled clinic visits and telephone interviews; over a median of 78 months (range up to 22 years), no patient was lost to follow-up.

### 2.3 Statistical Analysis

Continuous variables with a normal distribution are reported as mean ± standard deviation and compared by two-sample *t*-test. Non-normally distributed continuous data are presented as median (interquartile range) and analyzed using the Mann–Whitney U test. Categorical data are reported as number (percentage) and compared with Pearson’s χ^2^ test when every expected cell count is ≥5, with the Yates-corrected χ^2^ test for 2 × 2 tables containing any expected count <5, and with the two-sided Fisher exact test when an expected count falls below 1.

Predictors of in-hospital mortality were identified using logistic regression models. Initially, univariable analysis was performed to identify potential predictors during the perioperative period. Variables significant in univariable analysis (*p* < 0.05) were included in multivariable logistic regression models to fine-tune the predictive model and verify the proportional odds assumption.

For long-term follow-up mortality, Cox proportional hazards regression models were used. Univariable Cox regression analysis identified potential predictors, and significant variables (*p* < 0.05) were included in multivariable analysis to derive the best predictive model. The proportional hazards assumption was checked for each Cox regression analysis. Kaplan-Meier survival curves were generated and compared using the log-rank test. To assess potential temporal trends, we divided the study period into three intervals (1993–2003, 2004–2012 and 2013–2019) and performed survival analyses for each time period.

To mitigate potential selection bias and confounding, we conducted sensitivity analyses using inverse probability of treatment weighting (IPTW) and propensity score matching (PSM). PSM between the isolated and concomitant TVR groups was conducted using a multivariable logistic regression model with the following variables: age, sex, body mass index, hypertension, diabetes mellitus, chronic lung disease, chronic liver disease, chronic kidney disease, prior stroke, left ventricular ejection fraction, New York Heart Association (NYHA) Class III/IV, coronary artery disease, previous cardiac surgery, TRI-SCORE, peripheral vascular disease and right-sided heart failure signs. We employed a nearest neighbor matching algorithm with a 1:1 ratio for PSM and applied the same set of variables for IPTW to maintain consistency. The balance of covariates after PSM and IPTW was evaluated using standardized differences, with values below 0.1 indicating well-balanced groups.

To explore whether outcomes differ between first-time isolated TVR and redo isolated TVR, we conducted sensitivity analyses repeating all outcome models using a three-level exposure variable: (i) concomitant TVR (reference), (ii) primary-isolated TVR (no prior cardiac surgery), and (iii) redo-isolated TVR (≥1 prior cardiac surgery). Covariate adjustment was identical to the main analysis.

Statistical significance was set at a two-tailed *p*-value of <0.05. All analyses were conducted using R version 4.2.2 (R Core Team, Auckland, New Zealand) and Stata/MP version 17.0 (StataCorp LLC, College Station, TX, USA).

## 3. Results

### 3.1 Demographic and Clinical Characteristics

**Fig. 1.**
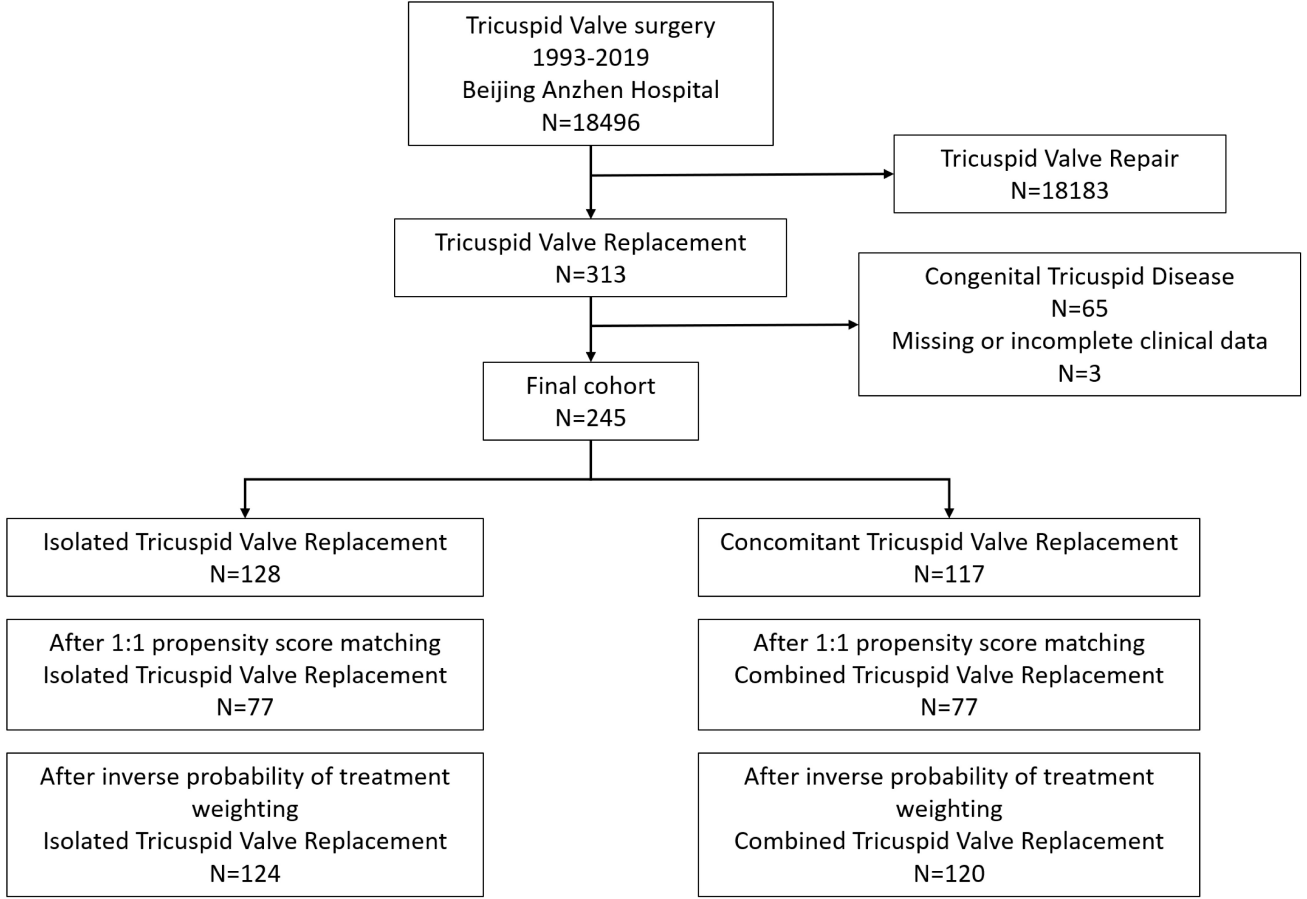
**Study cohort selection for tricuspid valve replacement analysis**. Flowchart showing the selection process of the study cohort for TVR at Beijing Anzhen Hospital from 1993 to 2019. The chart details the exclusions and final cohort distribution into isolated and combined TVR groups.

Between 1993 and 2019, a total of 18,496 patients were referred for tricuspid valve surgery at Beijing Anzhen Hospital. Out of these, 245 patients met the inclusion criteria and were included in this study (Fig. 1). Among them, 128 patients (52%) underwent isolated TVR, while 117 patients (48%) underwent TVR concomitant with other major procedures. Patients who underwent isolated TVR had a lower prevalence of male gender and a higher body mass index. They also had more instances of previous cardiac surgery and previous tricuspid valve surgery. Additionally, these patients exhibited lower EuroSCORE II and systolic pulmonary artery pressure compared to those undergoing concomitant TVR. The baseline characteristics of the study population are summarized in Table 1.

**Table 1.** **Baseline characteristics**.

	Isolated TVR (n = 128)	Concomitant TVR (n = 117)	*p* value
Age, y	47 ± 13	47 ± 12	0.793
Men	66 (51.6)	77 (65.8)	0.024
Body mass index, kg/m^2^	22.6 ± 3.5	21.7 ± 3.7	0.052
Hypertension	11 (8.6)	7 (6.0)	0.434
Diabetes mellitus	7 (5.5)	2 (1.7)	0.222
Chronic lung disease	3 (2.3)	2 (1.7)	1.000
Peripheral vascular disease	4 (3.1)	3 (2.6)	1.000
Prior stroke	2 (1.6)	3 (2.6)	0.919
Chronic liver disease	5 (3.9)	2 (1.7)	0.518
CKD	8 (6.2)	8 (6.8)	1.000
CAD	1 (0.8)	4 (3.4)	0.314
NYHA Class III/IV	76 (59.4)	78 (66.7)	0.238
Atrial fibrillation	74 (57.8)	82 (70.1)	0.046
Previous cardiac surgery	73 (57.0)	37 (31.6)	<0.001
Previous TV surgery	37 (28.9)	11 (9.4)	<0.001
Rheumatic	23 (18.0)	62 (53.0)	<0.001
Functional	53 (41.4)	32 (27.4)	0.021
Right-sided HF signs	61 (47.7)	65 (55.6)	0.217
Liver enlargements	31 (24.2)	39 (33.3)	0.115
Spleen enlargements	24 (18.6)	20 (17.1)	0.736
Ascites	24 (18.8)	18 (15.4)	0.485
Daily dose of loop diuretics, mg	20 (10–30)	20 (10–40)	0.845
EuroSCORE II	4.1 (1.8–6.3)	5.0 (2.6–9.2)	0.009
TRI-SCORE	3.8 ± 2.3	4.2 ± 2.2	0.105
Body surface area, m^2^	1.64 ± 0.16	1.59 ± 0.19	0.042
eGFR, mL/min	93 ± 34	85 ± 28	0.060
Hemoglobin, g/L	130 ± 25	128 ± 26	0.654
White blood cell, ×10^9^/L	5.58 ± 2.50	5.83 ± 2.61	0.452
Platelet, ×10^9^/L	155 ± 63	167 ± 73	0.160
BUN, mmol/L	6.4 (5.0–8.3)	6.3 (4.7–8.1)	0.601
Creatine, µmol/L	71.0 (59.8–80.4)	77.2 (62.1–80.4)	0.446
ALT, U/L	26 (20–35)	29 (23–38)	0.112
AST, U/L	22 (16–35)	23 (16–34)	0.726
Total bilirubin, µmol/L	25.0 ± 15.2	28.3 ± 19.0	0.135
Total protein, g/L	67.4 ± 10.8	67.3 ± 9.9	0.914
Albumin, g/L	40.0 ± 6.8	38.6 ± 6.0	0.094
LVEF, %	63 ± 10	62 ± 10	0.450
LVEDD, mm	44 ± 7	48 ± 9	<0.001
LVESD, mm	29 ± 6	32 ± 8	0.001
TAPSE, mm	17 ± 4	17 ± 4	0.400
RV basal diameter, mm	49 ± 12	48 ± 14	0.449
RA major dimension, mm	79 ± 20	78 ± 21	0.650
SPAP, mm Hg	44 ± 11	53 ± 18	<0.001

Values are number (percentage), mean ± standard deviation or median (interquartile range). TVR, tricuspid valve replacement; CKD, chronic kidney disease; CAD, coronary artery disease; TV, tricuspid valve; HF, heart failure; eGFR, estimated glomerular filtration rate; BUN, blood urea nitrogen; ALT, alanine aminotransferase; AST, aspartate aminotransferase; LVEF, left ventricular ejection fraction; LVEDD, left ventricular end-diastolic dimension; LVESD, left ventricular end-systolic dimension; TAPSE, tricuspid annular plane systolic excursion; RV, right ventricle; RA, right atrium; SPAP, systolic pulmonary artery pressure.

### 3.2 Surgical Information and In-Hospital Outcomes

Operative characteristics and clinical outcomes are presented in Table 2. The concomitant TVR group had significantly longer bypass time [153 (123–196) minutes versus 99 (76–133) minutes, *p* < 0.001] and cross-clamp time [99 (78–135) minutes versus 49 (0–66) minutes, *p* < 0.001] compared to the isolated TVR group. In the entire cohort, 97 patients (39.6%) received a mechanical prosthesis, with the most common valve sizes being 31 mm (used in 119 patients). Post-operatively, the isolated TVR group showed generally more favorable outcomes. In-hospital mortality was significantly lower than in the concomitant group (7.8% versus 17.9%, *p* = 0.017). Acute renal failure occurred less often, although this difference did not reach statistical significance (8.6% versus 16.2%, *p* = 0.068). Additionally, the isolated TVR group had shorter mechanical ventilation times [20 (14–38) hours versus 23 (16–48) hours, *p* = 0.016] and shorter intensive care unit (ICU) stays [37 (18–62) hours versus 44 (20–90) hours, *p* = 0.002].

**Table 2.** **Surgical information and clinical outcomes**.

		Isolated TVR (n = 128)	Concomitant TVR (n = 117)	*p* value
Emergent surgery		6 (4.7)	0	0.050
Concomitant surgery				
	Mitral valve repair	0	9 (7.7)	0.004
	Mitral valve replacement	0	78 (66.7)	<0.001
	Aortic valve replacement	0	26 (22.2)	<0.001
	CABG	0	4 (3.4)	0.109
	Aortic surgery	0	5 (4.3)	0.056
Surgical ablation		13 (10.2)	14 (12.0)	0.651
Bypass time, min		99 (76–133)	153 (123–196)	<0.001
Clamp time, min		49 (0–66)	99 (78–135)	<0.001
Mechanical valve		45 (35.2)	52 (44.4)	0.138
Valve size, mm		31 (29–31)	29 (29–31)	0.431
In-hospital mortality		10 (7.8)	21 (17.9)	0.017
Mechanical ventilation, h		20 (14–38)	23 (16–48)	0.016
Length of stay in ICU, h		37 (18–62)	44 (20–90)	0.002
Acute renal failure		11 (8.6)	19 (16.2)	0.068
Acute renal failure requiring dialysis		9 (7.0)	11 (9.4)	0.498
Bleeding		10 (7.8)	10 (8.5)	0.834
Liver failure		1 (0.8)	6 (5.1)	0.098
Perioperative stroke		0 (0.0)	2 (1.7)	0.227
Re-exploration		13 (10.2)	12 (10.3)	0.979
Follow-up, month		78 (26–111)	75 (2–121)	0.683
Overall mortality		29 (22.7)	47 (40.2)	0.003

Values are number (percentage), mean ± standard deviation or median (interquartile range). CABG, coronary artery bypass graft surgery; ICU, intensive care unit.

Factors associated with in-hospital mortality in univariate and multivariate analyses are presented in Table 3. In the univariate analysis, several variables were examined. In the univariate analysis, age, isolated TVR, TRI-SCORE, chronic kidney disease and signs of right-sided heart failure showed significant associations with in-hospital mortality. To mitigate the risk of overfitting in our multivariate analysis, we limited the number of predictors to three, based on the number of observed events (31 deaths). In the multivariate analysis, isolated TVR remained an independent protective factor for in-hospital mortality (OR = 0.40; 95% CI 0.17–0.95; *p* = 0.037).

**Table 3.** **Univariate and multivariate logistic regression for in-hospital death**.

	Univariate analysis		Multivariate analysis	
	OR (95% CI)	*p* value	OR (95% CI)	*p* value
Isolated TVR	0.39 (0.17–0.86)	0.020	0.40 (0.17–0.95)	0.037
TRI-SCORE	1.61 (1.32–1.98)	<0.001	1.55 (1.25–1.93)	<0.001
CKD	4.90 (1.64–14.62)	0.004	2.13 (0.61–7.41)	0.237

OR, odds ratio; CI, confidence intervals.

### 3.3 Long-Term Outcomes of Isolated TVR

**Fig. 2.**
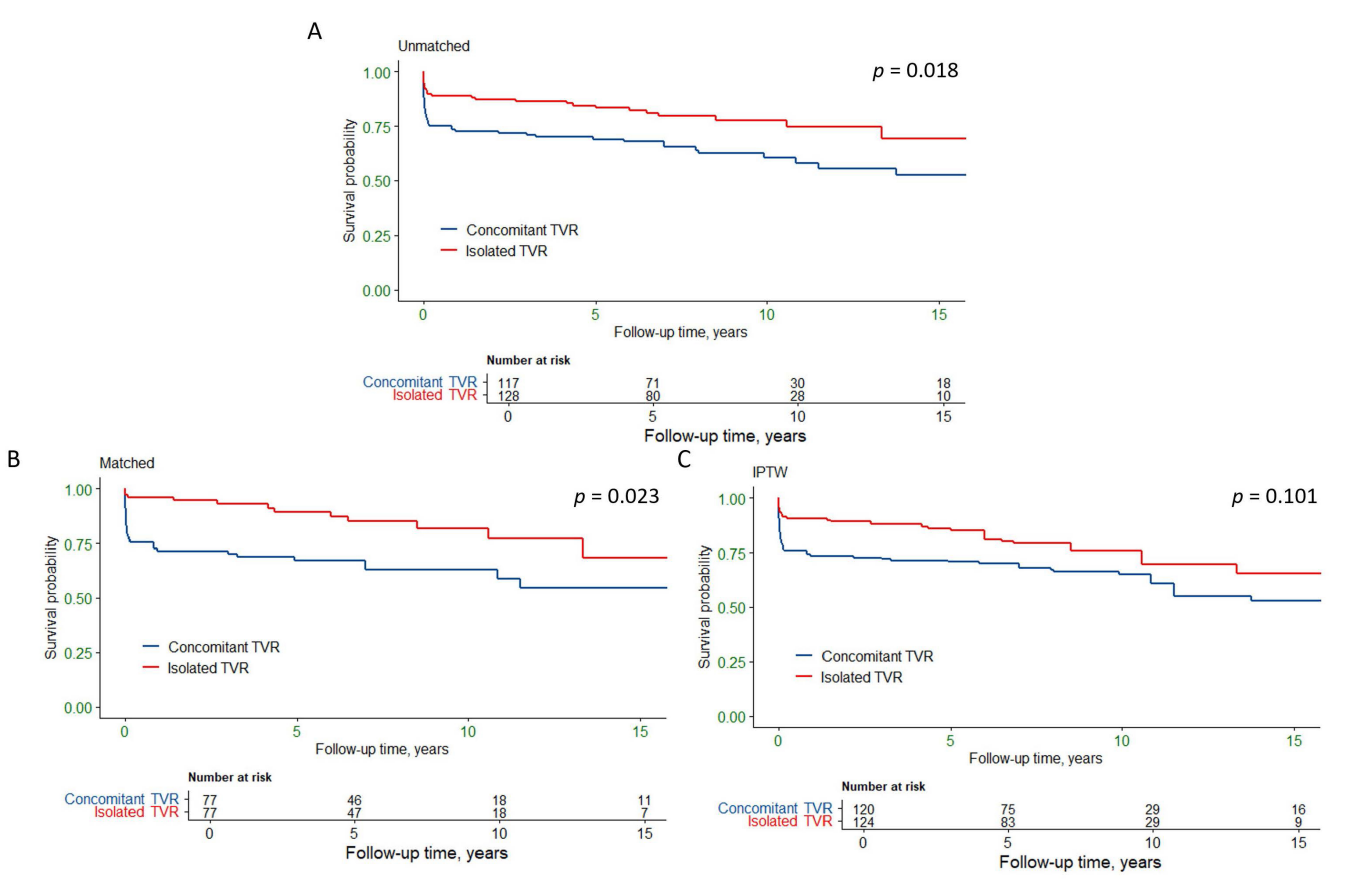
**Kaplan-Meier survival curves for isolated and concomitant tricuspid valve replacement**. (A) Unmatched analysis shows a significant difference in survival between the two groups (*p* = 0.018). (B) Propensity score matched analysis shows a significant difference in survival (*p* = 0.023). (C) Inverse probability of treatment weighting analysis indicates a trend towards lower mortality in the isolated TVR group (*p* = 0.101). TVR, tricuspid valve replacement; IPTW, Inverse probability of treatment weighting.

Follow-up data were available for all participants, with a mean follow-up duration of 6.8 ± 5.7 years [median 6.5 years (1.8–9.6), up to 21.9 years]. The isolated TVR group exhibited a significantly lower prevalence of overall mortality compared to the concomitant TVR group (*p* = 0.018, Fig. 2A). At 1, 5, and 10 years, the survival rates for isolated TVR were 89.1%, 83.3%, and 77.7%, respectively, while for concomitant TVR they were 72.6%, 68.9%, and 60.5%, respectively.

Univariate Cox regression analysis indicated that overall mortality was associated with age, isolated TVR, chronic kidney disease, TRI-SCORE and previous cardiac surgery history (Table 4). After adjusting for covariates, isolated TVR remained an independent protective factor for overall mortality (HR = 0.49, 95% CI: 0.30–0.81, *p* = 0.005).

**Table 4.** **Univariate and multivariate Cox regression for overall mortality**.

	Univariate analysis		Multivariate analysis	
	HR (95% CI)	*p* value	HR (95% CI)	*p* value
Isolated TVR	0.57 (0.36–0.91)	0.018	0.49 (0.30–0.81)	0.005
Age, years	1.04 (1.02–1.06)	<0.001	1.03 (1.01–1.05)	0.005
CKD	3.89 (2.04–7.46)	<0.001	1.82 (0.89–3.73)	0.101
TRI-SCORE	1.37 (1.23–1.52)	<0.001	1.32 (1.17–1.48)	<0.001
Previous cardiac surgery	1.84 (1.17–2.90)	0.009	1.74 (1.04–2.92)	0.034

HR, hazard ratio.

### 3.4 Overall Survival by Time Period

**Fig. 3.**
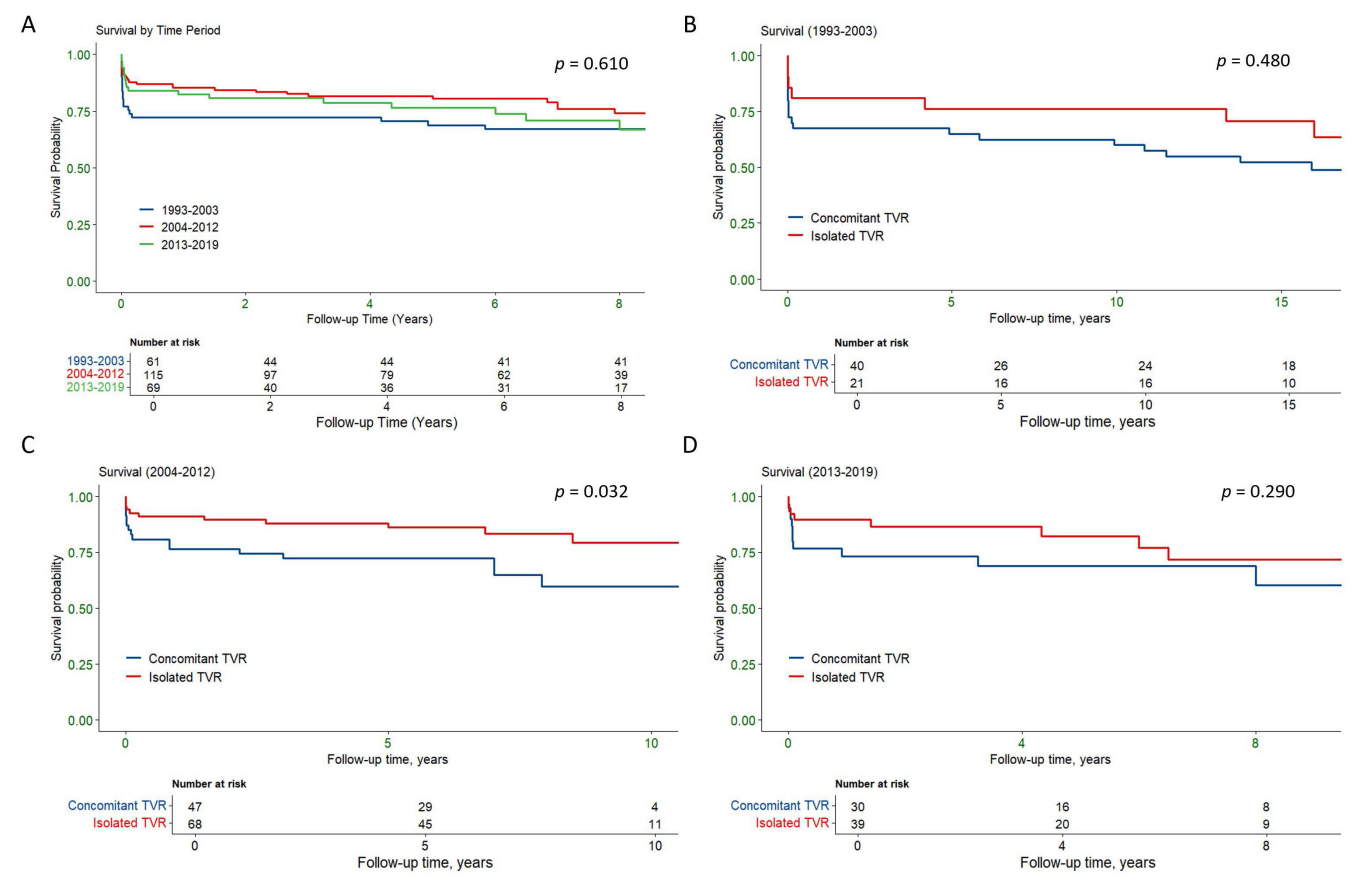
**Kaplan-Meier survival curves comparing overall survival by time period and between isolated and concomitant TVR groups**. (A) Overall survival across three time periods: 1993–2003, 2004–2012, and 2013–2019, showing no significant differences in survival across the time periods (*p* = 0.610). (B) Survival comparison between concomitant and isolated TVR in the 1993–2003 period, showing no significant difference (*p* = 0.480). (C) Survival comparison between concomitant and isolated TVR in the 2004–2013 period, with isolated TVR showing significantly better survival (*p* = 0.032). (D) Survival comparison between concomitant and isolated TVR in the 2014–2019 period, showing no significant difference (*p* = 0.290). TVR, tricuspid valve replacement.

The survival outcomes were analyzed across three time periods: 1993–2003, 2004–2012, and 2013–2019 to assess potential temporal trends (Fig. 3). Overall survival did not differ significantly across the time periods (*p* = 0.61, Fig. 3A). In the 1993–2003 period, no significant difference in survival was observed between concomitant and isolated TVR (*p* = 0.48, Fig. 3B). However, during the 2004–2012 period, isolated TVR was associated with significantly better survival compared to concomitant TVR (*p* = 0.032, Fig. 3C). In the 2013–2019 period, there was no significant survival difference between the two groups (*p* = 0.29, Fig. 3D). These results suggest that while there may have been a temporal effect in the 2004–2012 period favoring isolated TVR, no consistent trends were observed over the entire study period.

### 3.5 PSM and IPTW Analysis

To account for baseline differences and validate our findings, we conducted matching between patients in the isolated and concomitant TVR groups. After PSM, isolated TVR group showed significantly lower in-hospital mortality (3.9% versus 18.2%, *p* = 0.005) and long-term follow-up overall mortality (19.5% versus 37.7%, *p* = 0.013) compared to the concomitant TVR group. However, the smaller sample size post-PSM may have diminished the statistical power of these comparisons. To mitigate this limitation, we additionally applied IPTW using the same set of covariates as in PSM.

**Fig. 4.**
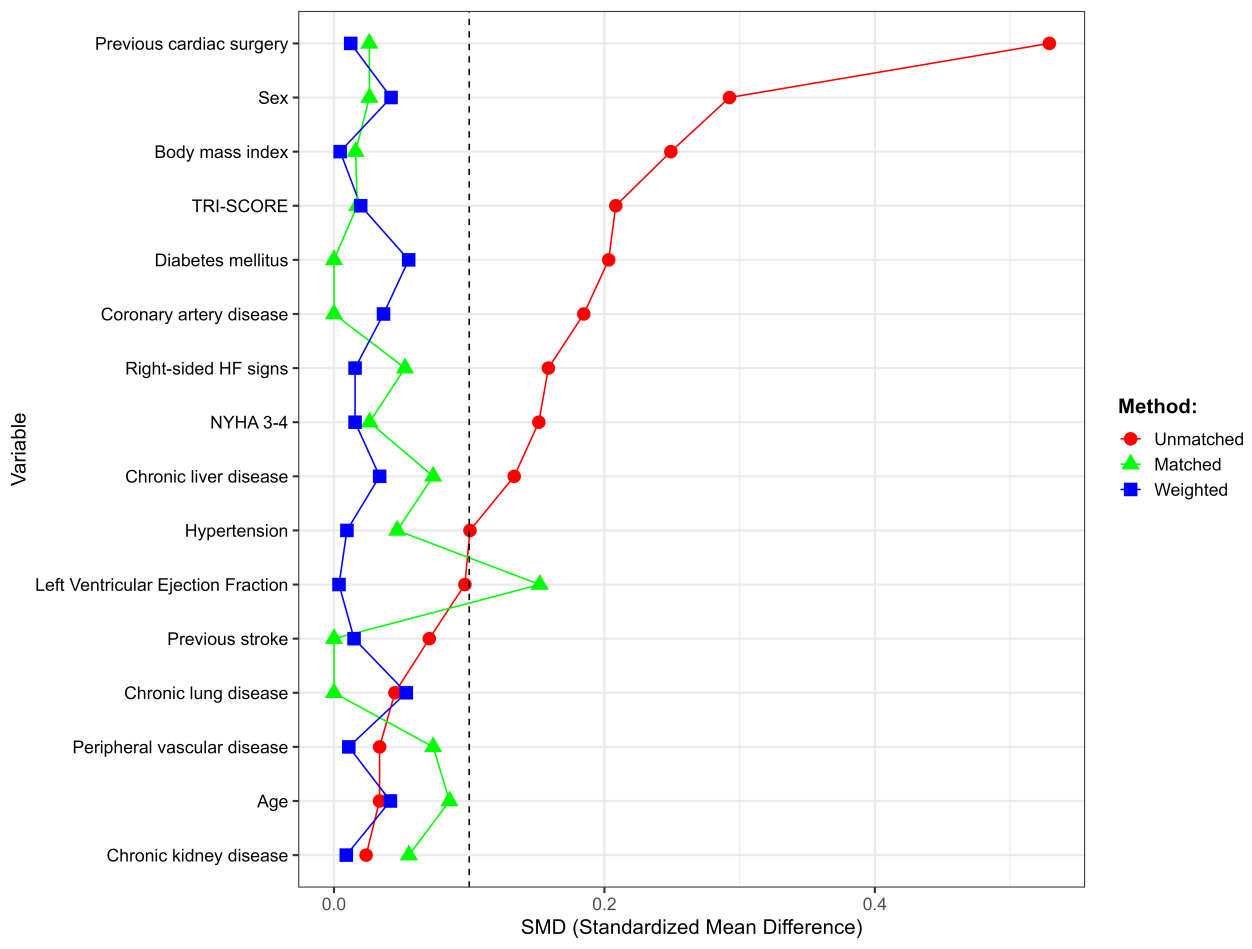
**Standardized mean differences for covariates across unmatched, matched, and weighted groups**. Standardized mean differences for various covariates in the unmatched, propensity score matched, and inverse probability of treatment weighting groups. The dashed line at SMD = 0.1 indicates the threshold for acceptable balance. The figure shows improved balance post-matching and weighting, with nearly all variables achieving SMD below the threshold.

After both PSM and IPTW adjustments, the standardized differences for nearly all covariates were below 0.1, except left ventricular ejection fraction for PSM, indicating good balance between the groups (Fig. 4). After IPTW, the isolated TVR group showed lower in-hospital mortality (6.6% versus 17.1%, *p* = 0.013) and long-term follow-up overall mortality (24.5% versus 37.0%, *p* = 0.064) than the concomitant TVR group. Table 5 presents logistic regression results for in-hospital mortality. Both PSM analysis and IPTW analysis confirms lower in-hospital mortality for isolated TVR [(OR = 0.09, 95% CI: 0.02–0.42, *p* = 0.003) and (OR = 0.28, 95% CI: 0.11–0.70, *p* = 0.007)]. Table 6 shows the Cox regression results for long-term overall mortality. Fig. 2B,C illustrate the survival for overall mortality in the PSM and IPTW analyses for the isolated and concomitant TVR groups. Detailed characteristics of the study cohort after PSM and IPTW are shown in **Supplementary Tables 1–4**.

**Table 5.** **Summary of in-hospital mortality and odds ratio for isolated TVR**.

		Sample size		In-hospital death	
		Isolated TVR	Concomitant TVR	OR (95% CI)	*p* value
Unmatched	Univariable	128	117	0.39 (0.17–0.86)	0.020
	Multivariable	128	117	0.40 (0.17–0.95)	0.037
Matched		77	77	0.09 (0.02–0.42)	0.003
Weighted		124	120	0.28 (0.11–0.70)	0.007

Matched/weighted factors: age, sex, body mass index, hypertension, diabetes mellitus, chronic lung disease, chronic liver disease, chronic kidney disease, prior stroke, left ventricular ejection fraction, New York Heart Association Class III/IV, coronary artery disease, previous cardiac surgery, TRI-SCORE, peripheral vascular disease and right-sided heart failure signs.

**Table 6.** **Summary of overall mortality and hazard ratio for isolated TVR**.

		Sample size		Overall death	
		Isolated TVR	Concomitant TVR	HR (95% CI)	*p* value
Unmatched	Univariable	128	117	0.57 (0.36–0.91)	0.018
	Multivariable	128	117	0.49 (0.30–0.81)	0.005
Matched		77	77	0.38 (0.20–0.74)	0.005
Weighted		124	120	0.56 (0.33–0.94)	0.028

Matched/weighted factors: age, sex, body mass index, hypertension, diabetes mellitus, chronic lung disease, chronic liver disease, chronic kidney disease, prior stroke, left ventricular ejection fraction, New York Heart Association Class III/IV, coronary artery disease, previous cardiac surgery, TRI-SCORE, peripheral vascular disease and right-sided heart failure signs.

### 3.6 Sensitivity Analysis

Among the 55 primary-isolated, 73 redo-isolated, and 117 concomitant cases, in-hospital deaths occurred in 0%, 13.7%, and 17.9%, respectively (two-sided Fisher’s exact *p* = 0.001). In the multivariable logistic model restricted to patients with at least one event (redo-isolated plus concomitant), TRI-SCORE remained independently associated with mortality (adjusted OR = 1.51, 95% CI 1.21–1.88; *p* < 0.001), whereas CKD and the redo-isolated indicator were not significant (**Supplementary Table 5**).

During follow-up, overall mortality was 10.9% (6/55), 31.5% (23/73) and 40.2% (47/117) in the primary-isolated, redo-isolated and concomitant cohorts, respectively (Fisher’s exact *p* < 0.001). Primary-isolated TVR conferred a significant long-term survival advantage over concomitant TVR (adjusted HR = 0.35, 95% CI 0.14–0.82; *p* = 0.017), whereas redo-isolated TVR showed no statistical difference (adjusted HR = 0.72, 95% CI 0.43–1.19; *p* = 0.194) (**Supplementary Tables 6,7**). A higher TRI-SCORE remained an independent predictor of late mortality (HR 1.28 per point, 95% CI 1.12–1.45; *p* < 0.001). **Supplementary Fig. 1** illustrate the survival for overall mortality in primary-isolated, redo-isolated and concomitant TVR.

## 4. Discussion

Over a 25-year span at our center, we retrospectively compared outcomes after TVR performed in isolation versus alongside other valve procedures. Even after adjusting for baseline risk factors—and confirming findings via multivariable modeling, PSM, and IPTW—patients who underwent isolated TVR experienced significantly lower perioperative and long-term mortality.This finding challenges the historical perception of isolated TVR as a high-risk salvage procedure and underscores the interplay among patient selection, surgical timing, and perioperative optimization.

Although TVR is relatively rare in cardiac valve surgeries, it carries a significantly high mortality rate [16]. Recent years have seen an increase in isolated TV surgeries due to greater recognition of the serious health risks posed by severe TR [11]. Despite this trend, there remains a lack of comprehensive data on the long-term mortality outcomes following TVR [17]. Current guidelines advocate for TV intervention, including both repair and replacement, during left-sided valve surgery for patients with severe symptomatic and asymptomatic TR [18]. For those with severe primary TR (stage C) who are asymptomatic but exhibit progressive right ventricular dilation or systolic dysfunction, isolated TV surgery is advised as a potential option [18]. Our findings challenge the traditional perception of isolated TVR as a higher-risk procedure by demonstrating comparable and, in some cases, better outcomes than concomitant TVR, especially with careful patient selection and optimized perioperative management. After adjusting for baseline risk, isolated TVR remained independently protective against perioperative death, while concomitant TVR was associated with higher in-hospital mortality, likely driven by the additional complexity of multi-valve operations and more advanced cardiac pathology in that group. At our center, tricuspid valve replacement was reserved for cases where repair was not feasible; thus, patients in the concomitant-TVR group generally presented with more advanced cardiac disease or required additional interventions, factors that inherently elevate perioperative risk. Conversely, those selected for isolated TVR at our center generally had preserved right ventricular function, minimal left-sided pathology, and manageable pulmonary pressures. By intervening before irreversible right-ventricular decompensation, we achieved better outcomes, echoing emerging literature that emphasizes early referral and optimized perioperative management to prevent adverse remodeling.

Several factors likely contribute to the survival advantage observed in isolated TVR. First, early referral and strict patient selection—favoring those with preserved right ventricular function, minimal left-sided pathology, and controlled pulmonary pressures—optimizes surgical tolerance. Second, contemporary surgical and perioperative advances (minimally invasive access, enhanced myocardial protection, precise valve sizing via advanced imaging, and tailored fluid management) reduce intraoperative stress and postoperative complications. Finally, dedicated postoperative pathways—with aggressive right-heart monitoring, early mobilization, and specialized ICU protocols—further mitigate morbidity. In contrast, concomitant TVR entails longer cardiopulmonary bypass times, increased procedural complexity, and a patient population with more advanced cardiac disease—factors known to elevate perioperative risk.

Our observations align with contemporary studies demonstrating that isolated TVR mortality rates have halved over the past decade through improved patient selection rather than technical factors alone. For example, Hamandi *et al*. [19] demonstrated that with advancements in surgical approaches and perioperative care, isolated TVR could be performed with lower operative mortality, reflecting our finding. These outcomes suggest that isolated TVR, when performed on appropriately selected patients, can lead to favorable outcomes with reduced postoperative morbidity. Similarly, Leviner *et al*. [8] emphasized that outcomes in high-risk cardiac surgeries, such as TVR, are strongly dependent on meticulous patient selection and perioperative management. Within our cohort, the greatest survival gains occurred during 2004–2012, a period marked by advancements in myocardial protection, ICU management protocols, and anticoagulation monitoring—although our retrospective design precludes isolating the impact of individual innovations.

Between 2004 and 2012, we observed a roughly 50% reduction in isolated TVR mortality, driven largely by enhanced patient selection and perioperative care rather than purely new surgical hardware. During this era, our center implemented blood-based cardioplegia with routine modified ultrafiltration, adopted semi-rigid annuloplasty rings and minimally invasive access, and rolled out fast-track ICU protocols emphasizing early extubation and goal-directed fluid management. Formal heart-team reviews and wider use of three-dimensional echocardiography further refined timing and candidate selection. In our cohort, the TRI-SCORE—an index incorporating age, renal impairment, liver dysfunction, New York Heart Association class, and left ventricular ejection fraction—was the single strongest predictor of mortality. Chronic kidney disease was similarly powerful. These findings underscore the need for detailed, patient-level risk stratification—beyond surgical strategy alone—to guide optimal timing and perioperative management in TVR candidates. Additionally, a higher-than-average 32% of patients received mechanical prostheses—reflecting our younger, largely rheumatic population and supported by a structured anticoagulation program that maintained a 72% time-in-therapeutic-range with zero valve thromboses. Taken together, these findings reinforce the value of early, isolated TVR in well-selected patients, guided by multidisciplinary evaluation and tailored perioperative pathways, and point to the need for future prospective trials to pinpoint which specific innovations yield the greatest survival benefit.

Our sensitivity analysis further refines this message. When the isolated cohort was split into primary-isolated and redo-isolated procedures, only the primary-isolated group retained a clear prognostic advantage. By contrast, redo-isolated TVR—performed after prior left-sided surgery—showed intermediate, non-significant outcomes. The slightly higher—although statistically non-significant—in-hospital mortality seen in the concomitant cohort (17.9% vs 13.7%) is probably driven by the additional hemodynamic burden and operative complexity of multivalve surgery (longer bypass times, more advanced left-sided disease, higher pulmonary pressures) rather than by the tricuspid procedure itself. These data suggest that the survival benefit of “isolated” TVR is time-dependent: it is greatest when tricuspid replacement is undertaken before multivalve disease and right-heart remodeling accrue, and it fades once patients have undergone previous cardiac operations. Clinically, this supports earlier referral for first-time tricuspid intervention within a multidisciplinary framework, while underscoring that redo-isolated cases should be counselled about an outlook closer to concomitant surgery. Importantly, TRI-SCORE remained the dominant predictor across all sub-groups, reinforcing the need to pair surgical timing with rigorous, patient-specific risk stratification.

### Limitations

This study has several limitations. First, the research was conducted at a single institution which may limit the generalizability of the findings to other settings or populations. Second, the retrospective design of the study may introduce selection bias, despite the use of PSM and IPTW to mitigate this issue. Third, the study included only patients who underwent TVR, excluding those who had tricuspid valve repair, which may limit the applicability of the results to the broader population of patients with tricuspid valve disease. The relatively small sample size (245 patients) might affect the statistical power of the study and the robustness of the conclusions drawn. Formal anatomic staging of tricuspid disease was not available in this retrospective cohort, necessitating reliance on surrogate markers and TRI-SCORE for disease-severity assessment. Additionally, detailed cause-specific mortality data were not available in our retrospective cohort, preventing formal adjudication of operative deaths. Detailed rates of prosthetic valve failure were unavailable in this retrospective series, limiting our ability to assess long-term valve durability. Furthermore, the 25-year study period encompasses significant advancements in surgical techniques and postoperative care, introducing variability based on the treatment era. Lastly, the heterogeneity in patient populations regarding previous cardiac surgeries may result in residual confounding, despite our efforts to adjust for these factors. Addressing these limitations in future research will be crucial to corroborate our findings and enhance the understanding of TVR outcomes.

## 5. Conclusion

In conclusion, our study demonstrates that isolated TVR is associated with lower in-hospital and long-term mortality compared to concomitant TVR. Importantly, the presence of chronic kidney disease and a higher TRI-SCORE emerged as consistent predictors of adverse outcomes across both timeframes, underscoring the need to address these comorbidities during preoperative risk stratification and postoperative care. These findings highlight the need for early intervention and careful patient selection to optimize outcomes in tricuspid valve surgery. Further research is essential to refine surgical strategies and improve prognosis for patients with tricuspid regurgitation, particularly in high-risk subgroups.

## Data Availability

The data that support the findings of this study are available from the corresponding author upon reasonable request.

## Abbreviations

TVR, tricuspid valve replacement; PSM, propensity score matching; IPTW, inverse probability of treatment weighting; TR, tricuspid regurgitation; TV, tricuspid valve; NYHA, New York Heart Association; OR, odds ratio; HR, hazard ratio.

## Supplementary Material

Supplementary material associated with this article can be found, in the online version, at https://doi.org/10.31083/RCM38102.
