# Supplementary material for: Isolated and Concomitant Tricuspid Valve Replacement: Long-Term Survival and Predictors of Mortality in a 25-Year Cohort
Source: Rev Cardiovasc Med. 2025 Nov 11;26(11):38102. doi: 10.31083/RCM38102 (PMC12681014; doi:10.31083/RCM38102)
Supplement: Supplementary file 1 [file 2153-8174-26-11-38102-s1.docx]

| **Supplementary Table 1 Baseline characteristics after propensity matching** | | | |
| --- | --- | --- | --- |
|  | **Isolated TVR**  **(n = 77)** | **Concomitant TVR**  **(n = 77)** | ***P* Value** |
| Age, y | 47 ± 13 | 48 ± 13 | 0.597 |
| Men | 44 (57.1) | 43 (55.8) | 0.871 |
| Body mass index, kg/m^2^ | 22 ± 3 | 22 ± 4 | 0.921 |
| Hypertension | 6 (7.8) | 7 (9.1) | 0.772 |
| Diabetes mellitus | 2 (2.6) | 2 (2.6) | 1 |
| Chronic lung disease | 2 (2.6) | 2 (2.6) | 1 |
| Peripheral vascular disease | 2 (2.6) | 3 (3.9) | 1 |
| Prior stroke | 2 (2.6) | 2 (2.6) | 1 |
| Chronic liver disease | 3 (3.9) | 2 (2.6) | 1 |
| CKD | 5 (6.5) | 4 (5.2) | 1 |
| CAD | 1 (1.3) | 1 (1.3) | 1 |
| NYHA Class III/IV | 44 (57.1) | 45 (58.4) | 0.870 |
| Atrial fibrillation | 42 (54.5) | 48 (62.3) | 0.327 |
| Previous cardiac surgery | 35 (45.5) | 34 (44.2) | 0.871 |
| Previous TV surgery | 18 (23.4) | 10 (13.0) | 0.095 |
| Rheumatic | 14 (18.2) | 31 (40.3) | 0.003 |
| Functional | 32 (41.6) | 29 (37.7) | 0.621 |
| Right-sided HF signs | 34 (44.2) | 32 (41.6) | 0.745 |
| Liver enlargements | 17 (22.1) | 16 (20.8) | 0.844 |
| Spleen enlargements | 17 (22.1) | 11 (14.3) | 0.210 |
| Ascites | 14 (18.2) | 9 (11.7) | 0.258 |
| Daily dose of loop diuretics, mg | 20 (10-30) | 20 (10-30) | 0.777 |
| EuroSCORE II | 3.8 (1.3-5.5) | 5.7 (2.3-11.7) | 0.001 |
| TRI-SCORE | 3.7 ± 2.4 | 3.7 ± 2.1 | 0.915 |
| Body surface area, m^2^ | 1.63 ± 0.14 | 1.63 ± 0.19 | 0.839 |
| eGFR, mL/min | 91 ± 32 | 91 ± 29 | 0.94 |
| Hemoglobin, g/L | 131 ± 25 | 132 ± 27 | 0.804 |
| White blood cell, ×10^9^/L | 5.74 ± 2.95 | 5.79 ± 2.23 | 0.913 |
| Platelet, ×10^9^/L | 159 ± 65 | 167 ± 67 | 0.566 |
| BUN, mmol/L | 6.3 (5.0-8.5) | 6.4 (4.6-8.2) | 0.655 |
| Creatine, μmol/L | 71 (57-80) | 77 (62-80) | 0.381 |
| ALT, U/L | 25 (20-34) | 28 (23-39) | 0.205 |
| AST, U/L | 24 (16-35) | 28 (16-36) | 0.827 |
| Total bilirubin, μmol/L | 25.4 ± 15.2 | 27.9 ± 20.2 | 0.375 |
| Total protein, g/L | 68 ± 9 | 66 ± 10 | 0.423 |
| Albumin, g/L | 40 ± 6 | 38 ± 6 | 0.039 |
| LVEF | 64 ± 10 | 63 ± 8 | 0.542 |
| LVEDD, mm | 43 ± 7 | 46 ± 7 | 0.023 |
| LVESD, mm | 29 ± 5 | 31 ± 8 | 0.045 |
| RV basal diameter, mm | 50 ± 13 | 48 ± 14 | 0.348 |
| RA major dimension, mm | 79 ± 21 | 76 ± 19 | 0.45 |
| TAPSE, mm | 18 ± 4 | 18 ± 4 | 0.966 |
| SPAP, mm Hg | 42 ± 11 | 50 ± 14 | <0.001 |
| Values are number (percentage), mean ± standard deviation or median (interquartile range).  TVR = tricuspid valve replacement; CKD = chronic kidney disease; CAD = coronary artery disease; TV = tricuspid valve; HF = heart failure; eGFR = estimated glomerular filtration rate; BUN = blood urea nitrogen; ALT = alanine aminotransferase; AST = aspartate aminotransferase; LVEF = left ventricular ejection fraction; LVEDD = left ventricular end-diastolic dimension; LVESD = left ventricular end-systolic dimension; TAPSE, tricuspid annular plane systolic excursion; RV = right ventricle; RA = right atrium; SPAP = systolic pulmonary artery pressure. | | | |

| **Supplementary Table 2 Surgical information and clinical outcomes after propensity matching** | | | |
| --- | --- | --- | --- |
|  | **Isolated TVR**  **(n = 77)** | **Concomitant TVR**  **(n = 77)** | ***P* Value** |
| Emergent surgery | 6 (7.8) | 0 | 0.037 |
| Concomitant surgery |  |  |  |
| Mitral valve repair | 0 | 7 (9.1) | 0.02 |
| Mitral valve replacement | 0 | 45 (58.4) | <0.001 |
| Aortic valve replacement | 0 | 19 (24.7) | <0.001 |
| CABG | 0 | 1 (1.3) | 1 |
| Aortic surgery | 0 | 5 (6.5) | 0.069 |
| Surgical ablation | 8 (10.4) | 9 (11.7) | 0.797 |
| Bypass time, min | 97 (77-118) | 146 (115-194) | <0.001 |
| Clamp time, min | 52 (35-68) | 92 (71-126) | <0.001 |
| Mechanical valve | 26 (33.8) | 34 (44.2) | 0.186 |
| Valve size | 31 (29-31) | 31 (29-31) | 0.155 |
| In-hospital mortality | 3 (3.9) | 14 (18.2) | 0.005 |
| Mechanical ventilation, h | 18 (13-28) | 22 (16-48) | 0.004 |
| Length of stay in ICU, h | 28 (16-65) | 44 (20-99) | 0.01 |
| Acute renal failure | 4 (5.2) | 16 (20.8) | 0.004 |
| Acute renal failure requiring dialysis | 3 (3.9) | 9 (11.7) | 0.071 |
| Bleeding | 5 (6.5) | 8 (10.4) | 0.385 |
| Liver failure | 0 | 5 (6.5) | 0.069 |
| Perioperative stroke | 0 | 1 (1.3) | 1 |
| Re-exploration | 5 (6.5) | 10 (13.0) | 0.174 |
| Follow-up, month | 80 (27-111) | 73 (10-114) | 0.281 |
| Overall mortality | 15 (19.5) | 29 (37.7) | 0.013 |
| Values are number (percentage), mean ± standard deviation or median (interquartile range).  TVR = tricuspid valve replacement; CABG = coronary artery bypass graft surgery; ICU = intensive care unit. | | | |

| **Supplementary Table 3 Baseline characteristics after inverse probability of treatment weighting** | | | |
| --- | --- | --- | --- |
|  | **Isolated TVR**  **(n = 124.5)** | **Concomitant TVR**  **(n = 119.7)** | ***P* Value** |
| Age, y | 48 ± 13 | 48 ± 12 | 0.771 |
| Men | 69.7 (56.0) | 69.6 (58.1) | 0.775 |
| Body mass index, kg/m^2^ | 22.3 ± 3.3 | 22.3 ± 3.8 | 0.975 |
| Hypertension | 10.7 (8.6) | 10.6 (8.8) | 0.951 |
| Diabetes mellitus | 5.7 (4.6) | 6.9 (5.8) | 0.769 |
| Chronic lung disease | 3.0 (2.4) | 4.0 (3.3) | 0.747 |
| Peripheral vascular disease | 3.8 (3.0) | 3.4 (2.9) | 0.936 |
| Prior stroke | 2.1 (1.7) | 2.3 (1.9) | 0.906 |
| Chronic liver disease | 3.9 (3.1) | 4.5 (3.8) | 0.829 |
| CKD | 7.6 (6.1) | 7.0 (5.9) | 0.944 |
| CAD | 1.8 (1.5) | 2.3 (1.9) | 0.8 |
| NYHA Class III/IV | 75.9 (61.0) | 73.9 (61.7) | 0.916 |
| Atrial fibrillation | 70.1 (56.3) | 78.3 (65.4) | 0.216 |
| Previous cardiac surgery | 58.0 (46.6) | 55.1 (46.0) | 0.933 |
| Previous TV surgery | 27.7 (22.3) | 18.6 (15.5) | 0.292 |
| Rheumatic | 24.8 (19.9) | 55.3 (46.2) | <0.001 |
| Functional | 48.2 (38.7) | 43.7 (36.5) | 0.761 |
| Right-sided HF signs | 60.4 (48.5) | 59.0 (49.3) | 0.914 |
| Liver enlargements | 30.0 (24.1) | 35.6 (29.7) | 0.369 |
| Spleen enlargements | 23.3 (18.7) | 20.7 (17.3) | 0.796 |
| Ascites | 25.3 (20.4) | 13.9 (11.6) | 0.08 |
| Daily dose of loop diuretics, mg | 20 (10-30) | 20 (10-30) | 0.658 |
| EuroSCORE II | 3.9 (1.4-5.8) | 5.8 (2.6-11.4) | <0.001 |
| TRI-SCORE | 3.8 ± 2.3 | 3.8 ± 2.2 | 0.894 |
| Body surface area, m^2^ | 1.62 ± 0.16 | 1.63 ± 0.20 | 0.741 |
| eGFR, mL/min | 92 ± 32 | 90 ± 29 | 0.656 |
| Hemoglobin, g/L | 130 ± 25 | 129 ± 26 | 0.799 |
| White blood cell, ×10^9^/L | 5.47 ± 2.51 | 5.87 ± 2.62 | 0.299 |
| Platelet, ×10^9^/L | 157 ± 62 | 162 ± 67 | 0.368 |
| BUN, mmol/L | 6.2 (4.9-8.2) | 6.2 (4.6-8.2) | 0.627 |
| Creatine, μmol/L | 69.0 (57.0-80.4) | 72.8 (62.1-80.4) | 0.181 |
| ALT, U/L | 26 (20-35) | 30 (23-38) | 0.037 |
| AST, U/L | 23 (16-35) | 25 (16-38) | 0.368 |
| Total bilirubin, μmol/L | 24.5 ± 14.4 | 27.4 ± 18.1 | 0.171 |
| Total protein, g/L | 67 ± 11 | 67 ± 10 | 0.778 |
| Albumin, g/L | 40 ± 7 | 39 ± 6 | 0.152 |
| LVEF | 63 ± 10 | 63 ± 9 | 0.977 |
| LVEDD, mm | 44 ± 7 | 48± 9 | <0.001 |
| LVESD, mm | 29 ± 6 | 31 ± 7 | 0.001 |
| RV basal diameter, mm | 49 ± 12 | 47 ± 14 | 0.352 |
| RA major dimension, mm | 80 ± 21 | 76 ± 19 | 0.188 |
| TAPSE, mm | 17 ± 4 | 18± 4 | 0.529 |
| SPAP, mm Hg | 43 ± 11 | 52± 16 | <0.001 |
| Values are number (percentage), mean ± standard deviation or median (interquartile range).  TVR = tricuspid valve replacement; CKD = chronic kidney disease; CAD = coronary artery disease; TV = tricuspid valve; HF = heart failure; eGFR = estimated glomerular filtration rate; BUN = blood urea nitrogen; ALT = alanine aminotransferase; AST = aspartate aminotransferase; LVEF = left ventricular ejection fraction; LVEDD = left ventricular end-diastolic dimension; LVESD = left ventricular end-systolic dimension; TAPSE, tricuspid annular plane systolic excursion; RV = right ventricle; RA = right atrium; SPAP = systolic pulmonary artery pressure. | | | |

| **Supplementary Table 4 Surgical information and clinical outcomes after inverse probability of treatment weighting** | | | |
| --- | --- | --- | --- |
|  | **Isolated TVR**  **(n = 124.5)** | **Concomitant TVR**  **(n = 119.7)** | ***P* Value** |
| Emergent surgery | 5.7 (4.6) | 0 | 0.019 |
| Concomitant surgery |  |  |  |
| Mitral valve repair | 0 | 10.4 (8.7) | 0.003 |
| Mitral valve replacement | 0 | 73.2 (61.1) | <0.001 |
| Aortic valve replacement | 0 | 27.8 (23.2) | <0.001 |
| CABG | 0 | 2.3 (1.9) | 0.049 |
| Aortic surgery | 0 | 6.2 (5.2) | 0.029 |
| Surgical ablation | 12.2 (9.8) | 11.6 (9.7) | 0.981 |
| Bypass time, min | 95 (74-125) | 151 (117-198) | <0.001 |
| Clamp time, min | 47 (0-65) | 94 (71-131) | <0.001 |
| Mechanical valve | 41.8 (33.6) | 48.9 (40.9) | 0.288 |
| Valve size | 31 (29-31) | 29 (29-31) | 0.196 |
| In-hospital mortality | 8.2 (6.6) | 20.4 (17.1) | 0.013 |
| Mechanical ventilation, h | 19 (14-36) | 23 (16-58) | 0.016 |
| Length of stay in ICU, h | 34 (18-63) | 43 (20-90) | 0.007 |
| Acute renal failure | 10.0 (8.0) | 21.9 (18.3) | 0.028 |
| Acute renal failure requiring dialysis | 7.8 (6.3) | 12.9 (10.8) | 0.255 |
| Bleeding | 10.8 (8.7) | 10.2 (8.5) | 0.972 |
| Liver failure | 0.7 (0.6) | 6.2 (5.2) | 0.013 |
| Perioperative stroke | 0 | 1.7 (1.4) | 0.184 |
| Re-exploration | 12.7 (10.2) | 12.7 (10.6) | 0.922 |
| Follow-up, month | 80 (31-111) | 75 (7-115) | 0.58 |
| Overall mortality | 30.4 (24.5) | 44.3 (37.0) | 0.064 |
| Values are number (percentage), mean ± standard deviation or median (interquartile range).  TVR = tricuspid valve replacement; CABG = coronary artery bypass graft surgery; ICU = intensive care unit. | | | |

| **Supplementary Table 5 Univariate and multivariate logistic regression for in-hospital death (redo-isolated vs concomitant, n = 190)** | | | | |
| --- | --- | --- | --- | --- |
|  | **Univariate analysis** | | **Multivariate analysis** | |
|  | **OR (95% CI)** | ***P* Value** | **OR (95% CI)** | ***P* Value** |
| Isolated TVR | 0.73 (0.32-1.64) | 0.442 | 0.61 (0.25-1.46) | 0.268 |
| TRI-SCORE | 1.61 (1.32-1.98) | <0.001 | 1.51 (1.21-1.88) | <0.001 |
| CKD | 4.90 (1.64-14.62) | 0.004 | 2.03 (0.57-7.23) | 0.274 |
| TVR = tricuspid valve replacement; OR = odds ratio; CI = confidence intervals; CKD, chronic kidney disease. | | | | |

| **Supplementary Table 6 Univariate and multivariate Cox regression for overall mortality (primary-isolated vs concomitant, n = 172)** | | | | |
| --- | --- | --- | --- | --- |
|  | **Univariate analysis** | | **Multivariate analysis** | |
|  | **HR (95% CI)** | ***P* Value** | **HR (95% CI)** | ***P* Value** |
| Isolated TVR | 0.25 (0.11-0.58) | 0.001 | 0.35 (0.14-0.82) | 0.017 |
| Age, years | 1.04 (1.02-1.06) | <0.001 | 1.05 (1.02-1.07) | <0.001 |
| CKD | 3.89 (2.04-7.46) | <0.001 | 1.70 (0.74-3.93) | 0.213 |
| TRI-SCORE | 1.37 (1.23-1.52) | <0.001 | 1.35 (1.17-1.55) | <0.001 |
| TVR = tricuspid valve replacement; HR = hazard ratio; CI = confidence intervals; CKD = chronic kidney disease. | | | | |

| **Supplementary Table 7 Univariate and multivariate Cox regression for overall mortality (redo-isolated vs concomitant, n = 190)** | | | | |
| --- | --- | --- | --- | --- |
|  | **Univariate analysis** | | **Multivariate analysis** | |
|  | **HR (95% CI)** | ***P* Value** | **HR (95% CI)** | ***P* Value** |
| Isolated TVR | 0.84 (0.51-1.39) | 0.501 | 0.72 (0.43-1.19) | 0.194 |
| Age, years | 1.04 (1.02-1.06) | <0.001 | 1.04 (1.02-1.06) | 0.001 |
| CKD | 3.89 (2.04-7.46) | <0.001 | 1.73 (0.84-3.57) | 0.135 |
| TRI-SCORE | 1.37 (1.23-1.52) | <0.001 | 1.28 (1.12-1.45) | <0.001 |
| TVR = tricuspid valve replacement; HR = hazard ratio; CI = confidence intervals; CKD = chronic kidney disease. | | | | |

**Supplementary
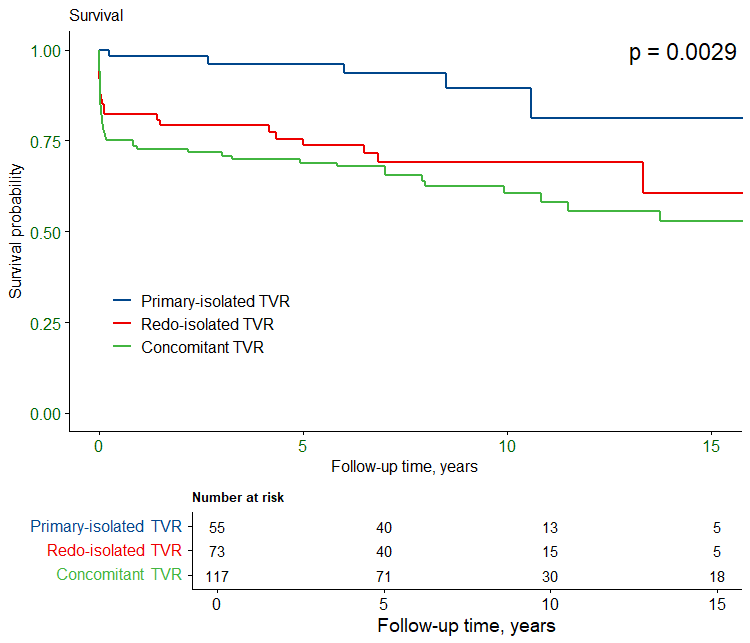
Fig. 1.** Kaplan–Meier survival curves for primary-isolated, redo-isolated and concomitant TVR (log-rank overall P = 0.0029). TVR = tricuspid valve replacement.
